# Dasatinib and quercetin mitigate radiation-induced lung injury by eliminating senescent cells in a rat model

**DOI:** 10.3389/fphar.2026.1748788

**Published:** 2026-02-27

**Authors:** Jing Liu, Xue Ren, Hengjiao Wang, Defu Yang, Ying Yan, Ying Xu

**Affiliations:** 1 Graduate School of Dalian Medical University, Dalian, China; 2 Department of Radiation Oncology, General Hospital of Northern Theater Command, Shenyang, China

**Keywords:** cellular senescence, dasatinib and quercetin, DNA damage, radiation-induced lung injury, senescence-associated secretory phenotype

## Abstract

**Background:**

Radiation-induced lung injury (RILI) is a major dose-limiting toxicity in thoracic radiotherapy, and accumulating evidence implicates radiation-induced cellular senescence in its pathogenesis. This study aimed to investigate whether combination therapy with dasatinib and quercetin (DQ) could mitigate RILI by reducing senescent cell burden.

**Methods:**

A rat model of RILI was established using a single 30 Gy irradiation to the right lung. Pulmonary pathological changes, fibrosis, DNA damage, and cellular senescence were assessed by histology, immunofluorescence, senescence-associated β-galactosidase staining, Western blotting and immunohistochemistry. Transcriptomic profiling was performed to explore the underlying molecular mechanisms.

**Results:**

Compared with irradiation alone, DQ treatment significantly alleviated radiation-induced inflammatory cell infiltration and collagen deposition, reduced γH2AX levels, decreased senescence-associated markers p53, p21, and p16, and suppressed multiple senescence-associated secretory phenotype (SASP) factors. Transcriptomic analysis indicated that DQ-mediated effects were closely associated with activation of apoptotic pathways and modulation of p53, MAPK, PI3K-Akt and mitophagy signaling cascades.

**Conclusion:**

DQ attenuated RILI in rats, with effects consistent with the reduced radiation-induced senescent cells and suppression of senescence-associated inflammatory responses.

## 1 Introduction

Radiotherapy is one of the three traditional pillars of cancer treatment. When combined with surgery, chemotherapy, or immunotherapy, it exhibits remarkable efficacy in controlling localized tumors (Kaur et al., 2023). However, while radiation therapy effectively eradicates malignant cells, it unavoidably exposes adjacent normal tissues to radiation, resulting in various toxic side effects (Cao et al., 2024). This occurs because radiation energy is inevitably deposited in the surrounding healthy tissues within the irradiation field during tumor targeting (Wang and Tepper, 2021). RILI is a common dose-limiting toxicity of thoracic radiotherapy, and its pathogenesis involves multiple factors, including DNA damage, inflammation, and fibrosis (Zhang X. et al., 2025). RILI can develop within hours to months after irradiation and is characterized by injury to alveolar epithelial and vascular endothelial cells, accompanied by inflammatory cell infiltration and aggregation (Wang Y. et al., 2024; Zhang M. et al., 2025).

Recent studies have demonstrated that ionizing radiation is closely associated with various age-related diseases and may even act as a potential inducer of premature aging (Al-Jumayli et al., 2022). Exposure to ionizing radiation induces a cascade of physiological alterations, leading to DNA double-strand breaks (DSBs), genomic instability, and metabolic dysfunction (Ibragimova et al., 2024). The biological effects of ionizing radiation vary with dose: high-dose exposure can cause acute radiation syndrome, whereas chronic low-dose exposure typically results in long-term pathological alterations (Wang et al., 2021). Cellular senescence is a permanent state of cell-cycle arrest triggered by stressors such as DNA damage and oxidative stress (Ahmad et al., 2024). In the context of RILI, radiation-induced cellular senescence has been recognized as a critical pathogenic mechanism (Zhu et al., 2024). Ionizing radiation can induce cell cycle arrest, disrupt autophagy homeostasis, and accelerate tissue aging (Li et al., 2023; Ren et al., 2023). Senolytic drugs are a novel class of therapeutic agents capable of reducing the burden of radiation-induced senescent cells without impairing the function of normal cells (Khosla et al., 2022). Dasatinib, a small-molecule tyrosine kinase inhibitor, can trigger apoptosis in certain senescent cell types (Kirkland and Tchkonia, 2020). Quercetin, a natural flavonoid compound, exerts senolytic effects by inhibiting key anti-apoptotic molecules within the BCL-2 family and related survival pathways in senescent cells (Chaib et al., 2022). The combination of DQ is currently among the most extensively studied senolytic regimens.

In summary, cellular senescence plays a pivotal role in the pathogenesis of RILI, and the targeted clearance of senescent cells represents a highly promising therapeutic strategy. However, whether senolytic drugs such as DQ can confer protective effects in RILI remains unclear. In this study, we established a rat model of RILI to characterize radiation-induced cellular senescence and to evaluate whether DQ intervention could effectively mitigate pulmonary damage. Our findings aim to enhance the mechanistic understanding of RILI and provide preclinical evidence supporting further evaluation of senolytic approaches for the prevention and treatment of RILI.

## 2 Materials and methods

### 2.1 Grouping and irradiation of laboratory animals

Male Sprague–Dawley rats (6–8 weeks old) were used to minimize potential variability associated with sex-related hormonal fluctuations. Rats were randomly divided into four groups (n = 3 per group): control (Con), drug-only (Con + DQ), irradiation (IR), and irradiation plus drug (IR + DQ). For DQ treatment, rats received dasatinib (5 mg/kg) and quercetin (50 mg/kg) by oral gavage once daily for five consecutive days, starting on day 1 post irradiation. All experimental procedures were approved by the Animal Care Committee of the General Hospital of Northern Theatre Command (Approval No. 2025-52) and were conducted in strict accordance with institutional animal welfare guidelines. Rats were housed in groups under controlled environmental conditions (24 °C ± 2 °C; 12-h light/dark cycle) with free access to food and water. Before irradiation, the animals were positioned under a CT simulator, and images were transferred to a treatment planning system to delineate the target area. Right-lung irradiation was performed using 6 MV X-rays at a dose rate of 300 cGy/min, delivering a single fraction of 30 Gy. After irradiation, rats were maintained under standard conditions, and lung tissues were harvested on day 28 for subsequent analyses.

### 2.2 Histopathological staining

Lung tissues were fixed in 4% paraformaldehyde, embedded in paraffin, and sectioned at 4 μm thickness. Hematoxylin–eosin (HE) staining was performed to evaluate general histopathological changes, while collagen deposition was evaluated using a Masson’s trichrome staining kit according to the manufacturer’s instructions.

### 2.3 Immunofluorescence

Frozen lung sections were thawed, fixed, permeabilized, and blocked with normal serum. Sections were incubated overnight at 4 °C with the γH2AX antibody (9718, 1:400, CST, United States). After washing with PBS, sections were incubated with fluorescently labeled secondary antibodies for 1 h at room temperature, counterstained with DAPI, and mounted with an anti-fade reagent. Images were acquired using a fluorescence microscope.

### 2.4 Senescence-associated β-galactosidase (SA-β-gal) staining

Frozen sections were thawed and washed three times with PBS (5 min each). Sections were fixed in β-galactosidase staining fixative for 15 min, washed again, and incubated with β-galactosidase staining working solution overnight at 37 °C. Observation under an optical microscope.

### 2.5 Western blotting

Lung tissues were lysed in RIPA buffer, and protein concentrations were determined using a BCA assay kit. Equal amounts of protein were separated by SDS-PAGE and transferred onto nitrocellulose membranes. Membranes were blocked with 5% skim milk for 2 h and then incubated overnight at 4 °C with primary antibodies, followed by incubation with HRP-conjugated secondary antibodies for 2 h at room temperature. Protein bands were visualized using an enhanced chemiluminescence (ECL) detection system. The following primary antibodies were used: p53 (SC126,1:500, Santa Cruz, United States), p21 (1:1000; ab109199, Abcam, United States), p16 (PA5-20379, 1:1000, Invitrogen, United States), IL-6 (GB11117, 1:1000, Servicebio, China), IL-1β (AF4006,1:1000, Affinity, China), IL-18 (E3G8R,1:1000, CST, United States), TNF-α (SC52B83,1:500, Santa Cruz, United States), MMP2 (SC13595, 1:500, Santa Cruz, United States), MMP9 (SC13520, 1:500, Santa Cruz, United States), α-SMA (19245, 1:1000, CST,United States), α-Tubulin (2125, 1:1000, CST, United States) and β-actin (GB15003, 1:5000, Servicebio, China).

### 2.6 Immunohistochemistry

Paraffin-embedded lung tissue sections were deparaffinized, rehydrated, and subjected to antigen retrieval. Endogenous peroxidase activity was quenched with hydrogen peroxide, and non-specific binding was blocked with goat serum. Sections were incubated overnight at 4 °C with the indicated primary antibodies against TGF-β (YP-Ab-15967,1:200, UpingBio, China) and MCP-1 (HY-P81171,1:200, MCE, China), followed by incubation with HRP-conjugated secondary antibodies. Immunoreactivity was visualized using a standard DAB chromogenic detection system, and images were captured under a light microscope.

### 2.7 Transcriptomic sequencing

Total RNA was extracted from lung tissues obtained from three independent biological replicates per group (n = 3). RNA integrity and concentration were evaluated using an Agilent 2100 Bioanalyzer. mRNA was isolated using oligo (dT) magnetic beads, and sequencing libraries were prepared following standard Illumina protocols. Library concentration was quantified using the Qubit™ system and qPCR, and fragment size distribution was verified using a bioanalyzer. After quality control, high-throughput sequencing was performed on an Illumina platform. Differentially expressed genes (DEGs) were identified using DESeq2, with |log2 fold change| ≥1 and adjusted *P* value <0.05. Principal component analysis (PCA) was performed to assess global expression patterns and revealed no obvious batch-driven clustering. DEGs were further analyzed by Gene Set Enrichment Analysis (GSEA), Gene Ontology (GO), and Kyoto Encyclopedia of Genes and Genomes (KEGG) pathway enrichment analyses.

### 2.8 Statistical analysis

All statistical analyses were performed using GraphPad Prism 8.0 software. Data are presented as mean ± SD. Normality was assessed using the Shapiro–Wilk test prior to parametric analyses. Comparisons between two groups were performed using an unpaired two-tailed Student's t-test. For comparisons among multiple groups, one-way ANOVA was used, followed by Tukey’s *post hoc* test. For body weight changes over time, two-way repeated-measures ANOVA was performed, with group and time as factors, followed by Bonferroni’s *post hoc* test when appropriate. A *P*-value of <0.05 was considered statistically significant (*P < 0.05, **P < 0.01, ***P < 0.001).

## 3 Results

### 3.1 DQ attenuates radiation-induced histopathological damage in rat lung

**FIGURE 1:**
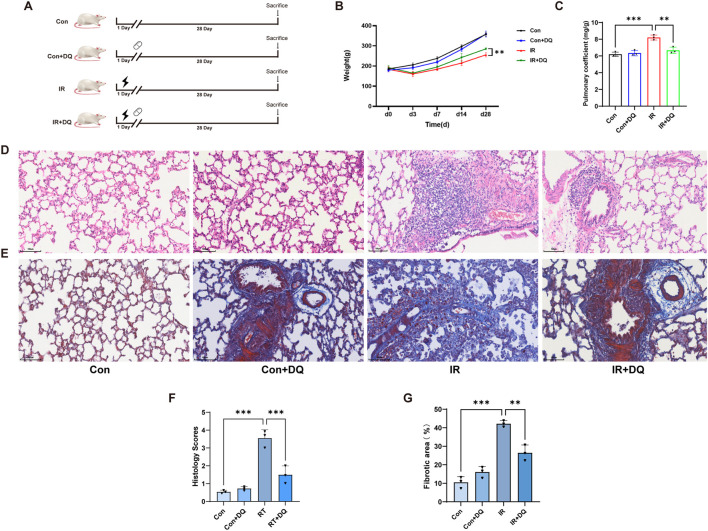
Pathological assessment of DQ-mediated protection against RILI. **(A)** Experimental grouping and schematic overview of the study design. **(B)** Body weight change of rats (n = 3). **(C)** The pulmonary coefficient in the different groups of rats (n = 3). **(D)** Representative images of HE staining (magnification ×200) in lung tissues. **(E)** Representative images of Masson’s trichrome staining (magnification ×200) in lung tissues. **(F)** Quantitative analysis of HE staining (n = 3). **(G)** Quantitative analysis of Masson’s staining (n = 3). Data are presented as mean ± SD. *P < 0.05, **P < 0.01, ***P < 0.001.

The experimental grouping and study design schematic is shown in Figure 1A. Irradiated rats exhibited a slower increase in body weight over time, which was partially reversed by DQ treatment. Consistently, the lung coefficient-an indicator of pulmonary edema-increased following irradiation, while DQ treatment markedly reduced this elevation (Figures 1B,C). The protective effects of DQ on RILI were assessed using HE and Masson’s trichrome staining. In the control group, HE staining revealed well-defined alveolar architecture, intact capillary networks, and the absence of congestion, edema, or inflammatory infiltration. In contrast, the IR group displayed severely thickened interalveolar septa, and extensive inflammatory cell infiltration. Quantitative analysis demonstrated that the histological injury score was significantly increased in the IR group (3.554 ± 0.478) compared with the control group (0.541 ± 0.085, *P* < 0.001). DQ treatment markedly alleviated RILI, reducing the histological score to 1.501 ± 0.495 in the IR + DQ group, corresponding to an approximately 57.8% decrease compared with the IR group (*P* < 0.001) (Figures 1D,F). Masson’s trichrome staining further revealed pronounced collagen accumulation after irradiation. Quantitative analysis showed that the collagen-positive area was significantly increased in the IR group (42.19 ± 1.68) compared with the control group (10.52 ± 3.02, P < 0.001). Importantly, DQ treatment significantly reduced collagen deposition to 26.46 ± 4.22 in the IR + DQ group, corresponding to an approximately 37.3% decrease relative to the IR group (*P* < 0.01) (Figures 1E,G). Collectively, these results demonstrated that combined DQ therapy effectively attenuates the histopathological progression of RILI in rats.

### 3.2 DQ attenuates radiation-induced DNA damage in rat lung

**FIGURE 2:**
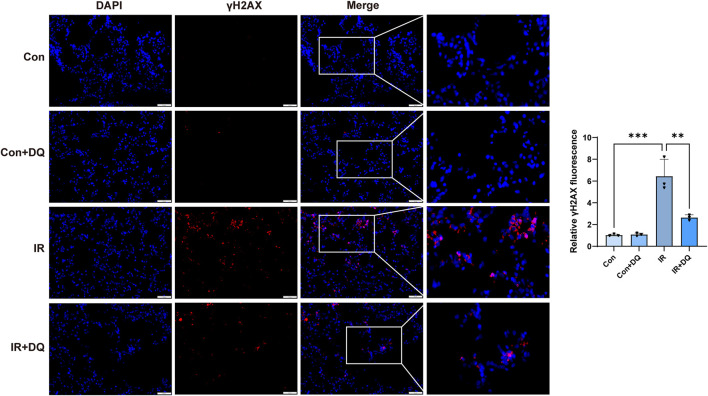
DQ attenuates radiation-induced DNA damage in lung tissues. Representative immunofluorescence staining images of γH2AX (red) and DAPI (blue) in lung tissues from each group (magnification ×200). Quantitative analysis of γH2AX fluorescence intensity is shown (n = 3). Data are presented as mean ± SD. *P < 0.05, **P < 0.01, ***P < 0.001.

Ionizing radiation induced extensive DNA damage. To evaluate the protective effect of DQ, the expression of the DNA damage marker γH2AX was examined in lung tissues from each group using immunofluorescence staining. In the IR group, abundant γH2AX-positive nuclear foci were observed, indicating pronounced DNA damage. Quantitative analysis showed that compared with the control group, the IR group exhibited a significant increase (6.370 ± 1.772 vs. 1.000 ± 0.006, *P* < 0.001). DQ treatment significantly reduced γH2AX signal intensity in irradiated lungs (2.708 ± 0.254 vs. 6.370 ± 1.772, *P* < 0.01), corresponding to an approximately 57.5% decrease relative to the IR group (Figure 2).

### 3.3 DQ alleviates radiation-induced pulmonary cellular senescence

**FIGURE 3:**
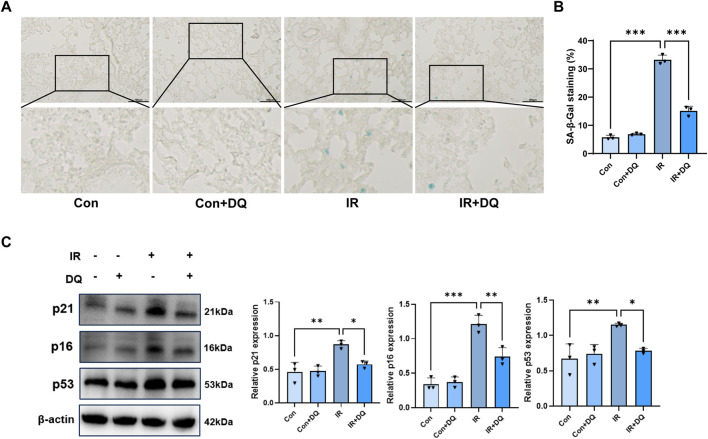
DQ treatment attenuates radiation-induced cellular senescence in rat lung tissue. **(A)** Representative images of SA-β-gal staining (magnification ×200). **(B)** Quantitative analysis of SA-β-gal staining (n = 3). **(C)** Representative Western blot images and quantitative analysis of p21, p16 and p53 (n = 3). Data are presented as mean ± SD. *P < 0.05, **P < 0.01, ***P < 0.001.

Persistent DNA damage can lead to tissue dysfunction and ultimately trigger cellular senescence. SA-β-gal staining was used as a primary indicator to evaluate cellular senescence. Quantitative analysis demonstrated a marked increase in SA-β-gal–positive cells in the IR group compared with controls (33.23 ± 1.67 vs. 5.74 ± 0.74, *P* < 0.001). DQ treatment significantly reduced senescent cell accumulation in irradiated lungs (15.11 ± 1.68 vs. 33.23 ± 1.67, *P* < 0.001), corresponding to an approximately 54.5% reduction relative to the IR group (Figure 3A). These results indicate that DQ treatment effectively mitigates radiation-induced cellular senescence. Western blot analysis further demonstrated that irradiation markedly upregulated the expression of key senescence-associated proteins in lung tissues. Specifically, p53 protein levels were significantly increased in the IR group compared with controls (1.151 ± 0.023 vs.0.669 ± 0.212, *P* < 0.01), whereas DQ treatment significantly reduced p53 levels by approximately 32.0% (0.783 ± 0.035 vs. 1.151 ± 0.023, *P* < 0.05). Similarly, p21 expression was significantly elevated following irradiation (0.867 ± 0.058 vs. 0.456 ± 0.145, *P* < 0.01), and DQ suppressed this increase by about 33.8% (0.574 ± 0.051 vs. 0.867 ± 0.058, *P* < 0.05). In parallel, p16 levels were substantially increased in the IR group relative to controls (1.213 ± 0.128 vs. 0.344 ± 0.091, *P* < 0.001), and DQ attenuated this upregulation by roughly 38.8% (0.742 ± 0.129 vs. 1.213 ± 0.128, *P* < 0.01) (Figures 3B,C). Collectively, these findings indicated at the molecular level that combined DQ therapy effectively attenuates radiation-induced cellular senescence in lung tissue.

### 3.4 Transcriptomic analysis reveals activation of apoptosis and metabolic reprogramming following DQ treatment

**FIGURE 4:**
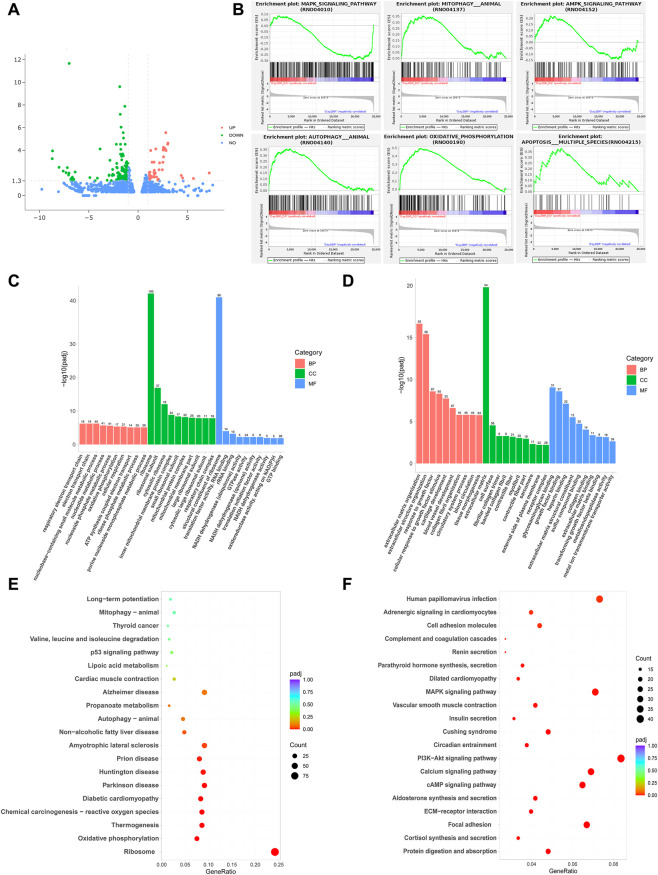
Transcriptomic analysis of DQ treatment in RILI. **(A)** DEGs between the IR and IR + DQ groups. **(B)** GSEA analysis. **(C,D)** GO enrichment analysis. **(E,F)** KEGG pathway enrichment analysis.

Transcriptomic sequencing was performed to further elucidate the molecular mechanisms underlying the protective effects of DQ against RILI, transcriptomic sequencing was performed to identify differentially expressed genes and signaling pathways involved in cellular senescence and apoptosis. The volcano plot of DEGs is presented in Figure 4A. GSEA identified significant differences in pathways related to cellular aging, including autophagy, apoptosis and mitophagy between the two groups (Figure 4B). These results suggested that DQ might exert its protective effects against RILI by modulating key signaling pathways involved in cellular stress and death. GO enrichment demonstrated the DEGs were predominantly enriched in oxidative phosphorylation under the biological process category, in mitochondrial-related components under the cellular component category, and in ATP synthase activity under the molecular function category (Figures 4C,D). KEGG pathway analysis further indicated that DQ treatment significantly affected multiple aging-related pathways, including the p53, MAPK, and PI3K-Akt signaling pathways, as well as mitophagy and oxidative stress responses (Figures 4E,F). These findings suggest that the senescence-clearing effects of DQ are closely associated with the regulation of apoptosis, mitochondrial metabolism, and oxidative stress pathways.

### 3.5 DQ effectively inhibits the SASP

**FIGURE 5:**
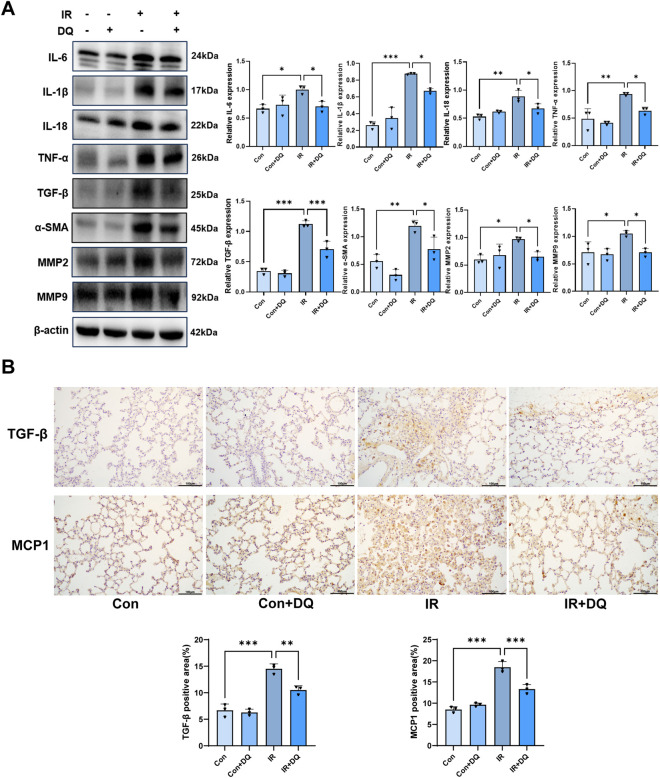
DQ suppresses radiation-induced SASP activation in lung tissue. **(A)** Representative Western blot images and quantitative analysis of IL-6, IL-1β, IL-18, TNF-α, TGF-β, α-SMA, MMP2, MMP9 (n = 3). **(B)** Representative immunohistochemical staining images (magnification ×200) and quantitative analysis of TGF-β and MCP1 expression in lung tissues (n = 3). Data are presented as mean ± SD. *P < 0.05, **P < 0.01, ***P < 0.001.

Senescent cells secrete various pro-inflammatory cytokines, chemokines, matrix metalloproteinases, and growth factors collectively known as the SASP. This phenotype has been reported to exert paracrine effects that promote senescence in neighboring normal cells (van Deursen, 2014; Hansel et al., 2020). Western blot analysis demonstrated that the expression levels of multiple SASP-associated inflammatory and fibrotic proteins were significantly increased in the IR group compared with the control group, including IL-6 (1.001 ± 0.072 vs. 0.666 ± 0.071, *P* < 0.05), IL-18 (0.885 ± 0.112 vs. 0.528 ± 0.047, *P* < 0.001), IL-1β (0.878 ± 0.009 vs. 0.262 ± 0.044, *P* < 0.001), TNF-α (0.938 ± 0.025 vs. 0.488 ± 0.185, *P* < 0.01), α-SMA (1.194 ± 0.102 vs. 0.556 ± 0.124, *P* < 0.01), TGF-β (1.123 ± 0.054 vs. 0.345 ± 0.051, *P* < 0.001), MMP2 (0.971 ± 0.038 vs. 0.602 ± 0.083, *P* < 0.05), and MMP9 (1.050 ± 0.052 vs. 0.706 ± 0.193, *P* < 0.05). Importantly, combined DQ treatment markedly attenuated the radiation-induced upregulation of these SASP components. Relative to the IR group, DQ treatment reduced IL-6 expression by approximately 30% (0.702 ± 0.092 vs. 1.001 ± 0.072, *P* < 0.05), IL-18 by 24% (0.673 ± 0.085 vs. 0.885 ± 0.112, *P* < 0.05), IL-1β by 24% (0.672 ± 0.031 vs. 0.878 ± 0.009, *P* < 0.05), TNF-α by 32% (0.636 ± 0.065 vs. 0.938 ± 0.025, *P* < 0.05), In parallel, profibrotic markers were also significantly suppressed, with α-SMA decreased by 35% (0.773 ± 0.220 vs. 1.194 ± 0.102, *P* < 0.05), TGF-β reduced by 37% (0.706 ± 0.131 vs. 1.123 ± 0.054, p < 0.001), Consistently, DQ treatment lowered the expression of matrix-remodeling enzymes, reducing MMP2 by 33% (0.654 ± 0.087 vs. 0.971 ± 0.038, *P* < 0.05), and MMP9 by 33% (0.705 ± 0.074 vs. 1.050 ± 0.052, *P* < 0.05). Collectively, these results indicated that ionizing radiation induced a robust SASP response in lung tissue, encompassing inflammatory, profibrotic, and extracellular matrix–remodeling components, while DQ treatment effectively suppresses this pathological SASP activation (Figure 5A) Immunohistochemical staining further corroborated these observations. Compared with controls, irradiation markedly increased TGF-β and MCP-1 expression in lung tissues (TGF-β: 14.51 ± 0.92 vs. 6.69 ± 1.19, *P* < 0.001; MCP-1: 18.48 ± 1.32 vs. 8.50 ± 0.68, *P* < 0.001). Following DQ treatment, TGF-β staining intensity was reduced by approximately 28% (10.52 ± 0.79 vs. 14.51 ± 0.92, P < 0.001), while MCP-1 expression decreased by 28% (13.32 ± 1.08 vs. 18.48 ± 1.32, P < 0.001) relative to the IR group (Figure 5B). These results further suggested that DQ eliminates radiation-induced senescent cells and mitigates SASP-associated profibrotic/proinflammatory responses in lung tissue.

## 4 Discussion

Radiotherapy is widely used to treat various solid tumors, including glioblastoma, lung cancer, breast cancer, rectal cancer, prostate cancer, colorectal cancer, cervical cancer, esophageal cancer, and head and neck malignancies (Larionova et al., 2022). Typical fractionated dose used in clinical radiotherapy range from 1.8 to 2.5 Gy, causing persistent damage to both tumor and normal cells, which consequently induces cellular senescence (Kim et al., 2023). Clinically, RILI often evolves from an early inflammatory pneumonitis phase to late fibrotic remodeling characterized by excessive extracellular matrix deposition and irreversible impairment of lung structure and function. RILI remains one of the most common and clinically significant complications limiting the efficacy and dosage of thoracic radiotherapy. Ionizing radiation causes irreversible cellular injury and aging through multiple mechanisms (Tong and Hei, 2020).

**FIGURE 6:**
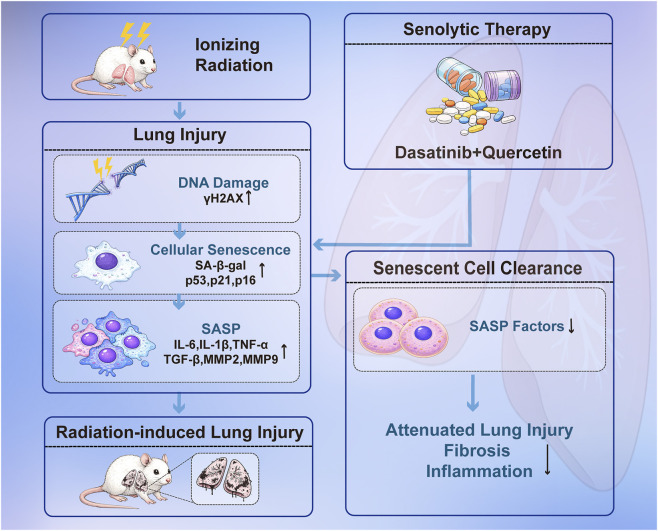
Schematic illustration of the senolytic effects of DQ on RILI.

In this study, we demonstrated that post-irradiation administration of DQ significantly attenuated RILI in a rat model, as evidenced by reduced inflammatory cell infiltration and collagen deposition together with improved lung architecture. A schematic diagram illustrating the mechanism by which DQ alleviates RILI is shown in Figure 6. Radiation-induced DNA damage is a key upstream trigger of cellular senescence. γH2AX is widely used as a marker of DNA double-strand break–associated damage responses (Valente et al., 2022). We also observed reduced γH2AX signaling following DQ treatment. This finding suggests that DQ eliminated senescent cells harboring persistent DNA damage foci rather than directly enhancing DNA repair—a mechanism consistent with senescence-drugging.

At the cellular and molecular levels, our data further elucidate the mechanisms underlying DQ’s efficacy. DQ treatment markedly reduced the burden of senescent cells, as shown by decreased SA-β-galactosidase activity and downregulation of the canonical senescence markers p53, p21, and p16. Cellular senescence is characterized by stable cell-cycle arrest, persistent DNA damage signaling, and altered secretory activity, and senescent cells in alveolar epithelial cells, macrophages, and fibroblasts have been identified as key contributors to radiation-induced pulmonary fibrosis (Soysouvanh et al., 2020; Su et al., 2021; Zhou et al., 2022; Wu et al., 2023). Our findings suggest the interpretation that DQ reduces the burden of radiation-induced senescent cells *in vivo*, thereby alleviating downstream tissue dysfunction.

A defining feature of senescent cells is the SASP, which comprises pro-inflammatory cytokines, chemokines, growth factors, and matrix-remodeling enzymes that reinforce chronic inflammation and fibrosis (Wang B. et al., 2024; Alqahtani et al., 2025). In the present study, DQ treatment significantly suppressed multiple SASP components, including IL-6, IL-1β, TNF-α, TGF-β, MCP-1, and matrix metalloproteinases. Given the established role of SASP in amplifying inflammatory and fibrotic responses in RILI, suppression of SASP may provide a mechanistic link between reduced senescence burden and the observed improvements in lung histopathology. These findings are consistent with previous reports describing the anti-aging and anti-fibrotic effects of DQ in multiple tissues. For instance, DQ reduces p16INK4A expression in aged cardiac tissue, partially reversing age-related cardiomyocyte hypertrophy and ventricular fibrosis (Lewis-McDougall et al., 2019). In genetically obese mice, DQ decreases senescent cell accumulation in liver tissue and ameliorates hepatic steatosis (Ogrodnik et al., 2017). Moreover, early clinical evidence supports the translational potential of DQ as a senolytic therapy—in a pilot clinical trial involving patients with idiopathic pulmonary fibrosis, DQ improved physical function and reduced tissue injury (Justice et al., 2019).

Transcriptomic analysis further revealed the mechanism of DQ’s therapeutic effect. The analysis revealed enrichment of apoptosis-related pathways following DQ treatment and regulation of associated signaling cascades such as p53, MAPK, and PI3K-Akt. These pathways are known to orchestrate DNA damage responses and control the balance between cell survival and programmed cell death, both of which are critical in determining the fate of radiation-damaged cells. The p53 pathway modulates the production and secretion of SASP components (Sheekey and Narita, 2023), while inhibition of p38 MAPK activity has been shown to delay cellular aging across multiple cell types (Goldman et al., 2023; Jie et al., 2025). Concurrently, the analysis revealed significant enrichment of mitochondrial metabolism and quality control pathways, including oxidative phosphorylation and mitophagy. This highlights a potential novel dimension of DQ action. Given the role of mitochondrial dysfunction in aging and SASP regulation (Victorelli et al., 2023), these findings provide new preliminary mechanistic insights into how DQ alleviates RILI and suggest a coordinated cellular response between nuclear stress signaling and mitochondrial reprogramming.

Several limitations of this study should be acknowledged. First, this study employed a single high-dose irradiation model, which does not fully replicate clinically fractionated radiotherapy regimens. Second, although our data indicate a marked reduction in the aging burden at the organizational level, the current study does not directly address the issue of aging selectivity at the single-cell or cell-type-specific level. Future studies employing fractionated irradiation, functional respiratory assessments, and single-cell or cell-type-resolved analyses will be valuable to strengthen the mechanistic and translational relevance of these findings.

In summary, this preclinical study demonstrated that post-irradiation DQ treatment alleviates RILI in rats. The protective effects are associated with a reduction in senescent cell burden, suppression of the pro-inflammatory and pro-fibrotic SASP, and modulation of associated apoptosis and mitochondrial pathways. These findings provide a mechanistic foundation for considering senolytic strategies, including DQ, as a potential therapeutic approach for RILI, while highlighting the need for further translational research.

## 5 Conclusion

In conclusion, this study demonstrated that post-irradiation treatment with DQ mitigates pathological features of RILI in rats. These protective effects were accompanied by reduced senescence-associated signals, decreased γH2AX burden, and broad suppression of SASP-related inflammatory and profibrotic factors. Collectively, these findings support the role of cellular senescence in promoting RILI development and provide preclinical rationale for further evaluating aging-targeted strategies in clinically relevant models.

## Data Availability

The datasets presented in this study can be found in online repositories. The names of the repository/repositories and accession number(s) can be found in the article/supplementary material.
