# Supplementary material for: Dasatinib and quercetin mitigate radiation-induced lung injury by eliminating senescent cells in a rat model
Source: Front Pharmacol. 2026 Feb 27;17:1748788. doi: 10.3389/fphar.2026.1748788 (PMC12982407; doi:10.3389/fphar.2026.1748788)
Supplement: Supplementary file 1 [file Supplementaryfile1.pdf]

Table 1. Original western blots

IL-6	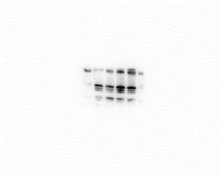	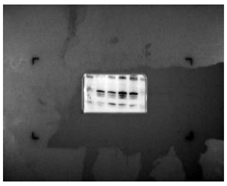
IL-1β	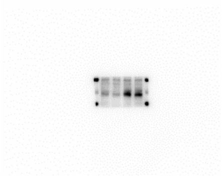	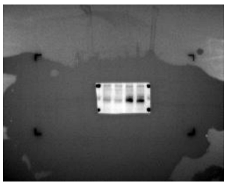
IL-18	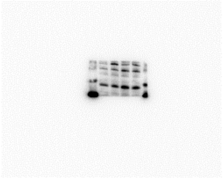	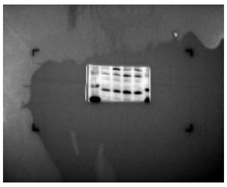
MMP2	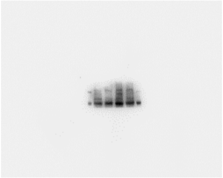	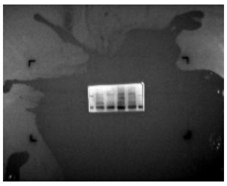
MMP9	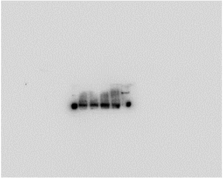	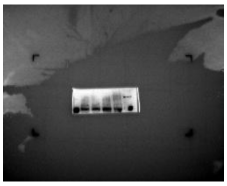
TNF-a	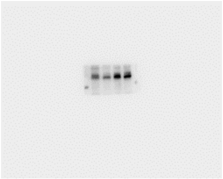	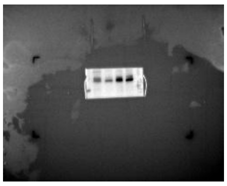
β-actin		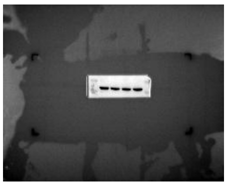
p16	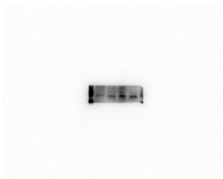	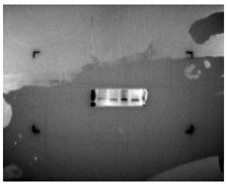

p21	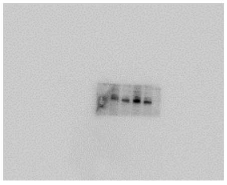	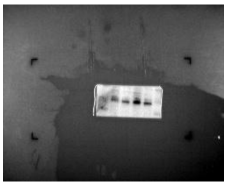
p53	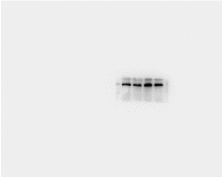	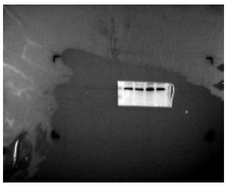
β-actin		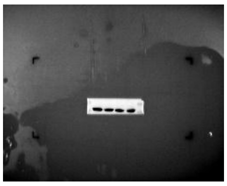
TGF-β	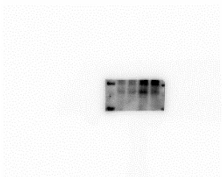	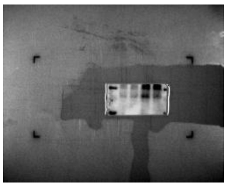
α-SMA	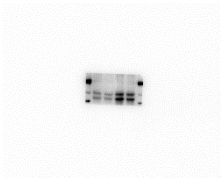	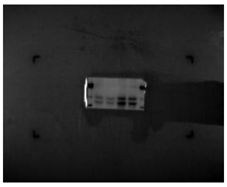
α-Tublin	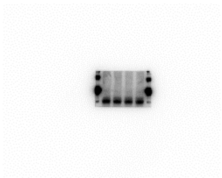	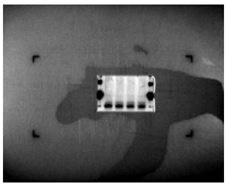
